# Reproducibility and Relative Validity of the Healthy Eating Index-2015 and Nutrient-Rich Food Index 9.3 Estimated by Comprehensive and Brief Diet History Questionnaires in Japanese Adults

**DOI:** 10.3390/nu11102540

**Published:** 2019-10-21

**Authors:** Kentaro Murakami, M. Barbara E. Livingstone, Aya Fujiwara, Satoshi Sasaki

**Affiliations:** 1 Department of Social and Preventive Epidemiology, School of Public Health, University of Tokyo, Tokyo 113-0033, Japan; fujiwaraay-tky@umin.ac.jp (A.F.); stssasak@m.u-tokyo.ac.jp (S.S.); 2 Nutrition Innovation Centre for Food and Health (NICHE), School of Biomedical Sciences, Ulster University, Coleraine BT52 1SA, UK; mbe.livingstone@ulster.ac.uk

**Keywords:** diet quality, HEI-2015, NRF9.3, Japan, dietary assessment questionnaire

## Abstract

We examined the reproducibility and relative validity of two measures of overall diet quality, the Healthy Eating Index-2015 (HEI-2015) and Nutrient-Rich Food Index 9.3 (NRF9.3), as estimated by well-established self-administered dietary assessment questionnaires for the Japanese, namely the comprehensive diet history questionnaire (DHQ) and the brief diet history questionnaire (BDHQ). Diet was assessed separately by two DHQs and two BDHQs at a 1-year interval and by 16-day weighed dietary records (DRs) in 121 women and 121 men aged 31–81 years. HEI-2015 and NRF9.3 were calculated from each method. The reproducibility correlation for the two questionnaires (intraclass correlation) ranged from 0.53 (HEI-2015 from BDHQ in men) to 0.77 (NRF9.3 from BDHQ in women). The validity correlation between the first questionnaires and DR (Pearson correlation) ranged from 0.37 (NRF9.3 from BDHQ in men) to 0.61 (NRF9.3 from DHQ and BDHQ in women). Bland–Altman plots showed poor agreement between the DHQ or BDHQ and DR, as well as the presence of weak proportional bias. Overall, these data indicate reasonable reproducibility and ranking ability of the DHQ and BDHQ for assessing the HEI-2015 and NRF9.3 and support their usefulness in future epidemiological research on the overall effects of Japanese diets on various health outcomes.

## 1. Introduction

Partly owing to the low prevalence of coronary artery disease and relatively good life expectancy of the Japanese, the diet consumed by the Japanese is widely perceived to be healthy [1,2,3]. However, empirical support for this assertion is limited, due to a lack of appropriate tools for assessing the overall quality of the Japanese diet. Studies have consistently determined that compliance with the Japanese healthy eating guidelines (Japanese Food Guide Spinning Top) is simultaneously associated with both favourable aspects of dietary intake (e.g., higher intake of dietary fibre and micronutrients) and unfavourable aspects (e.g., higher intake of saturated fats and sodium) [4,5,6,7]. Moreover, two large prospective cohort studies showed an inverse association between compliance with these guidelines and total mortality, in women at least [4,8].

In our recent systematic review of Japanese studies which obtained dietary patterns using a principal component analysis, we found that the food groups which contribute to dietary patterns termed healthy (vegetables, including potatoes, mushrooms, and seaweeds; fruits; soy products) are reasonably consistent with those often observed in Western countries [9]. Accordingly, diet quality measures which encompass a wide spectrum of dietary intake may be used to evaluate the quality of Japanese diets, even if the measure was developed for a Western dietary context. We speculated that the Healthy Eating Index-2015 (HEI-2015) [10,11,12] is a promising tool in this respect because, being based on general food groups in addition to three nutrients of worldwide concern (e.g., sodium, added sugars, and saturated fats), it is designed to assess adherence to the latest and highly comprehensive dietary guidelines (for Americans) [13] and uses a scoring system which is independent of the distribution of the studied population. The Nutrient-Rich Food Index 9.3 (NRF9.3), a composite measure of the nutrient density of the total diet [14,15,16,17,18], shares these characteristics, but is based on nutrient rather than food intake.

Accumulation of epidemiological evidence on the potential health effects of overall quality of the Japanese diet requires an assessment tool which is not only feasible and cost-effective but also accurate and reliable. To date, one comprehensive diet history questionnaire (DHQ) and a brief version (brief diet history questionnaire; BDHQ) have been well-established for this purpose, with sufficient validity at the food and nutrient level [19,20]. Here, we examined the reproducibility and relative validity of HEI-2015 and NRF9.3 estimated from the DHQ and BDHQ using a 4-day weighed dietary record (DR) conducted in each season over a 1-year period (16 days in total) as a reference method.

## 2. Materials and Methods

### 2.1. Data Source and Analytic Sample

The present study was based on data collected over one year (November 2002 to November 2003) in four geographically diverse areas in Japan: Osaka (Osaka City; urban), Okinawa (Ginowan City; urban island), Nagano (Matsumoto City; rural inland) and Tottori (Kurayashi City; rural coastal). Details of the study have been provided elsewhere [19,20,21,22]. In brief, we recruited apparently healthy women aged 30–69 years who were willing to participate and were living with their husbands. For each area, our recruitment strategy was to include eight women from each 10-year age category (30–39, 40–49, 50–59 and 60–69 years). Their husbands were then recruited (irrespective of their age), resulting in 128 individuals invited for each sex. Sample size was determined based primarily on the size of the sample of a previous validation study in Japan [23,24], in addition to feasibility. Inclusion criteria for this study were no self-reported major chronic disease (such as diabetes and cardiovascular disease) and community dwelling (free-living) individuals. Exclusion criteria were dietitian as profession, experience with dietary counselling from a doctor or dietitian, and history of hospitalisation for diabetes education. In total, 121 women and 121 men who completed the survey protocol were included in the analysis.

This survey was conducted according to the guidelines laid down in the Declaration of Helsinki and written informed consent was obtained from each individual participant. The use of the study data was approved by the University of Tokyo Faculty of Medicine Ethics Committee.

### 2.2. Dietary Record

As detailed previously [19,20,21,22], the participants completed the weighed DR on four non-consecutive days once in each of the four seasons at intervals of approximately three months, namely in November and December 2002 for autumn, February 2003 for winter, May 2003 for spring and August and September 2003 for summer. The sets of four recording days each comprised one weekend day and three weekdays within approximately two weeks. In the orientations, locally employed registered dietitians provided the participants with written and verbal instructions on how to maintain the recording and provided them with a sample completed record as an example. The couples were each provided with recording sheets and a digital scale (KD-173, Tanita, Tokyo, Japan; precision ±2 g at 0–250 g and ±4 g at 251–1000 g) and instructed on how each food and drink should be weighed. They were requested to document and weigh all foods and drinks taken on each of the recording days. On occasions when weighing was problematic (e.g., dining out), they were instructed to document as much information as possible, including the brand name of the food and the consumed portion size (based on typical household measures), as well as the details of leftovers. The participants were asked to fax the completed forms for each recording day to the local staff, who then reviewed the forms and, whenever necessary, sought additional information or modification of the record via telephone or fax. The responses were generally faxed to the centre, although some were handed directly to the centre staff. All the collected records were then reviewed by trained registered dietitians at each local centre, and again at the study centre. As requested in the study protocol, portion sizes estimated using household measures were converted into weights, and individual food items were coded based on the Standard Tables of Food Composition in Japan [25]. This food composition table is complete and widely used in Japan, containing nutrient content information for >2200 food codes. A total of 1392 food codes appeared in the DR. Estimated intakes of energy and selected nutrients for each day for each individual were calculated based on the intakes of food items and their nutrient contents. Added sugar intake was also calculated based on a recently compiled comprehensive composition database [26].

### 2.3. Diet History Questionnaires

In each season, the participants also answered the BDHQ and then DHQ approximately two days before the start of the dietary recording period (November 2002 for autumn, February 2003 for winter, May 2003 for spring and August and September 2003 for summer). Participants again answered the BDHQ and DHQ about one year after the first occasion (in November 2003). Responses to these questionnaires were checked for completeness by registered dietitians and when necessary, reviewed with the participant to ensure clarity of the answers.

Details of the structure and calculation procedures of the DHQ and BDHQ (as well as validity at the food and nutrient level) have been published elsewhere [19,20,21,27]. Briefly, the DHQ and BDHQ are structured self-administered questionnaires which assess dietary habits during the preceding month. They ask about the consumption frequency (and portion size in the DHQ) of selected foods commonly consumed in Japan, as well as general dietary behaviour and usual cooking methods. Standard portion sizes in the DHQ and fixed portion sizes in the BDHQ were derived mainly from several recipe books for Japanese dishes. Estimates of the daily intakes of foods (151 items in DHQ and 58 items in BDHQ), energy, and selected nutrients were calculated using an ad hoc computer algorithm based on the Standard Tables of Food Composition in Japan [28]. Added sugar intake was similarly calculated based on a recently compiled comprehensive composition database [26].

### 2.4. Calculation of HEI-2015

As described elsewhere [10,11,12], HEI-2015 is a composite measure of compliance with the 2015–2020 Dietary Guidelines for Americans [13]. These guidelines have been well validated in the US population [11,12]. HEI-2015 is a 100-point scale, with a higher score indicating a better quality of overall diet. The adequacy components (maximum score) are total fruits (5), whole fruits (5), total vegetables (5), greens and beans (5), whole grains (10), dairy (10), total protein foods (5), seafood and plant proteins (5) and fatty acids (ratio of the sum of polyunsaturated and monounsaturated fatty acids (in g) to saturated fatty acids (in g), 10). The moderation components are refined grains (10), sodium (10), added sugars (10) and saturated fats (10).

The scoring system of HEI-2015 is based on cup or ounce equivalents for food components, which are available for US foods (in the Food Patterns Ingredients Database (FPID) [29]) but not Japanese foods. Thus, a cup and ounce equivalent database for food items appearing in the Standard Tables of Food Composition in Japan [25] was prepared using the FPID. First, 289 of 2229 food items were identified as those containing no food components related to HEI-2015 (e.g., sugars, vegetable oils, and seasonings), for which the assignment of cup or ounce equivalents was not needed. Secondly, ounce equivalents for meat and seafood items (*n* = 715) were determined by the FPID definition of those for protein foods (1 ounce of cooked lean meat, poultry, or seafood can have no more than 2.63 g of allowable fat per 28.35 g of lean meat). Thirdly, for intact grains or grain products, such as rice (*n* = 122), 28.35 g of grains was defined as equal to 1 ounce grain equivalent (in accordance with FPID). Fourth, for fruit juice drinks (*n* = 19), the cup equivalent was determined based on the concentration of pure fruit juice as well as its cup equivalent (in accordance with FPID). Fifth, cup or ounce equivalent values were assigned for foods with a direct match in FPID (mainly fruits, vegetables, eggs, beans, legumes, dairy; *n* = 843). When there was no direct match, cup or ounce equivalent values of closely related food items in FPID were assigned (mainly Japanese vegetables and Japanese confectioneries, *n* = 204). For example, pickled turnip was assigned the value of raw turnip, while wheat-based Japanese cracker was assigned the value of pretzels. Finally, for other food items, such as konjac (*n* = 37), the cup or ounce equivalents were determined by the value of ingredients shown in FPID as well as the yield percentage.

The cup and ounce equivalent database for foods in the Standard Tables of Food Composition in Japan was then merged with the DR dataset (including 1392 food codes) and also incorporated into the calculation algorithm of the DHQ (including 236 food codes) and BDHQ (including 150 food codes). For each dietary assessment method, the HEI-2015 component and total scores were calculated based on energy-adjusted values (except for fatty acids), namely as cup or ounce equivalents (for food components) or amount (for sodium) per 4194 kJ (1000 kcal) of energy as well as percentage of energy (for added sugars and saturated fats).

### 2.5. Calculation of NRF9.3

The overall diet quality was also assessed using the NRF9.3, as described in detail elsewhere [13,14,15,16]. In short, the NRF9.3 is a validated composite measure of the nutrient density of the total diet, calculated as the sum of the percentage of reference daily values (RDVs) for nine qualifying nutrients minus the sum of the percentage of RDVs for three disqualifying nutrients. RDVs were determined (for sex and age categories) based on the Dietary Reference Intakes (DRIs) for Japanese, 2015 [30] (as shown in Table S1), namely the Recommended Dietary Allowance for protein, vitamins A and C, calcium, iron and magnesium, and tentative dietary goal for preventing lifestyle-related diseases for dietary fibre, potassium, saturated fats and sodium. In terms of added sugars, the conditional recommendation advocated by the World Health Organization (i.e., upper limit of 5% of energy) [31] was used because of the lack of a recommended value for added sugar in Japan as well as its low intake [26,32]. For each dietary assessment method, the NRF9.3 component and total scores were calculated as follows. The overall daily intake of each nutrient for each participant was adjusted for energy intake and then normalized for the sex- and age-specific Estimated Energy Requirement for a moderate level of physical activity (from DRIs) and expressed as a percentage of the RDV. For qualifying nutrients, the RDV percentage of each was terminated at 100 in order for a high intake of one nutrient not to compensate for the low intake of another. With regard to adverse nutrients, consideration was limited to the share which exceeded the recommended amount. Accordingly, higher NRF9.3 scores indicated a better quality of the overall diet, and a maximum possible score of 900 indicated a diet in which intakes per given amount of energy were above the RDVs for the nine qualifying nutrients but below the RDVs for the three disqualifying nutrients. In this study, dietary supplements were not considered during the nutrient intake calculation in any of the dietary assessment methods because it was our intention to assess nutrient intake from foods and beverages only.

### 2.6. Statistical Analysis

All statistical analyses were performed for women and men separately using Statistical Analysis System (SAS) statistical software (version 9.4, SAS Institute Inc, Cary, NC, USA). Since distribution of the total scores of HEI-2015 and NRF9.3 was not strongly skewed, data are presented as mean and standard deviation (SD). For the assessment of reproducibility of HEI-2015 and NRF9.3 (total and component scores) estimated by DHQ and BDHQ, we calculated intraclass correlation coefficients between dietary assessment questionnaires (DHQ or BDHQ) completed in the same season about one year apart, as well as differences in mean values (by paired *t*-test).

Preliminary scrutiny on the basis of each season’s dietary data showed relatively small variations in the total scores of HEI-2015 and NRF9.3 (differences in mean values within 7%; intraclass correlations 0.39–0.68) in any dietary assessment method (Table S2). Based on this, as well as considering that the single administration of a dietary assessment questionnaire is common practice in nutritional epidemiological studies, an assessment of relative validity was conducted based on comparison of a single DHQ or BDHQ with combined data of DRs covering all seasons (16 days in total; for each individual, the mean value of 16-day data was used). For this purpose, we used the first DHQ and BDHQ because the answers provided on other occasions (after the experience of conducting DR), but not the first DHQ and BDHQ (administered before the experience), may have been influenced by the attention to diet required to complete the DR. To assess the estimation ability at the group level, mean values of HEI-2015 and NRF9.3 derived from the DHQ or BDHQ were compared with those derived from the DR on the basis of paired *t*-test. Pearson correlation coefficients were used to assess the ability to rank the individuals in a population. We also calculated the percentage of subjects who were classified in the same, adjacent or opposite quintile of total diet quality scores in the two different assessment methods. In addition, agreement for the total scores of HEI-2015 and NRF9.3 between the DHQ or BDHQ and DR was assessed on the basis of the Bland–Altman plot [33] by plotting the difference in the scores (DHQ or BDHQ minus DR) against the average of both scores (estimate from DHQ or BDHQ plus that from DR divided by 2). A one-sample *t*-test was applied to assess the difference between the two measurements varied significantly from zero. The upper and lower limits of agreement were calculated as the mean difference ± 1.96 × SD. A linear regression was conducted to assess the presence of proportional bias. Finally, to investigate the dietary profiles associated with the overall diet quality, dietary intake of selected nutrients was calculated according to tertile category of the HEI-2015 or NRF9.3 (based on DR data). For this analysis, a *p* value for trend was calculated using a general linear model. A *p* value <0.05 was considered statistically significant.

All analyses were conducted not only based on the whole sample but also for each of the four areas separately. Since the separate analyses showed similar results (data not shown), we present the combined results here.

## 3. Results

The present analysis included 121 women aged 31–69 years and 121 men aged 31–81 years (Table 1). Mean energy intake estimated by the DHQ and BDHQ was significantly (*p* < 0.05) different from that estimated by DR, except for that by DHQ in men.

The HEI-2015 and NRF9.3 (component and total scores) calculated from each dietary assessment method are summarized in Table 2 and Table 3, respectively. In both diet quality measures, intraclass correlations between the two DHQs or two BDHQs (reproducibility correlations) for individual components varied considerably, ranging from 0.01 to 0.85 (median 0.56) in women and from −0.02 to 0.75 (median 0.50) in men. The mean values of individual components did not differ between the two DHQs or two BDHQs, with only a few exceptions.

Pearson correlations between the DR and DHQ or BDHQ (validity correlations) also varied considerably, with a range of 0.06 to 0.88 (median 0.45) in women and −0.02 and 0.68 (median 0.43) in men. The low correlations appeared mainly due to limited variation, including the presence of many participants with the maximum score (e.g., total protein foods, seafood and plant proteins and saturated fats for HEI-2015 and protein, vitamin D and saturated fats for NRF9.3). In many cases, the mean values of individual components significantly differed between the DR and DHQ or BDHQ.

The reproducibility correlation for the total score of HEI-2015 calculated by the DHQ was 0.58 in women and 0.57 in men (Table 2). The correlation based on the BDHQ was closely similar (0.57 in women and 0.53 in men). The corresponding correlation for the total score of NRF9.3 was 0.74 for the DHQ and 0.77 for the BDHQ in women and 0.61 for the DHQ and 0.56 for the BDHQ in men (Table 3). There was no significant difference between mean values of the total scores of HEI-2015 and NRF9.3 estimated by the two DHQs or two BDHQs.

Regarding the relative validity of the estimation ability at the group level, mean total scores of HEI-2015 estimated by the DHQ or BDHQ were significantly higher than those estimated by the DR, except for no difference for DHQ in men (Table 2). The mean total scores of NRF9.3 estimated by the DHQ were lower in both sexes, while that estimated by the BDHQ was higher in women, with no difference in men (Table 3). For the ability to rank the individuals in a population, Pearson correlation of the HEI-2015 total score estimated by the DHQ and BDHQ with that by the DR was 0.57 and 0.52 in women, respectively, and 0.51 and 0.43 in men, respectively (Figure S1). The percentage of subjects who were classified in the same, adjacent or opposite quintile of HEI-2015 derived from DR and those derived from DHQ were 34.7%, 40.5% and 0% in women and 35.5%, 39.7% and 0.02% in men, respectively. The corresponding percentage for BDHQ was 31.1%, 33.6% and 0.01% in women and 22.7%, 39.2% and 0.03% in men, respectively. The Pearson correlation with regard to NRF9.3 total score was 0.61 for both the DHQ and BDHQ in women and 0.55 for the DHQ and 0.37 for the BDHQ in men (Figure S2). The percentage of subjects who were classified in the same, adjacent or opposite quintile of NRF9.3 derived from DR and that derived from DHQ were 39.7%, 34.7% and 0.01% in women and 31.4%, 42.1% and 0.01% in men, respectively. The corresponding percentage for BDHQ was 28.1%, 38.8% and 0.01% in women and 23.1%, 42.1% and 0.02% in men, respectively.

**Figure 1:**
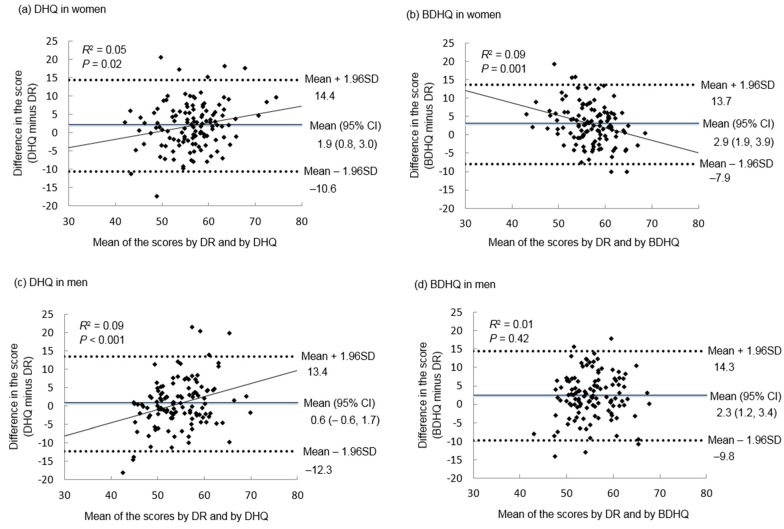
Bland–Altman plots assessing the agreement between the total score of Healthy Eating Index-2015 estimated by a 16-day weighed dietary record (DR) conducted over a year and that estimated by a comprehensive diet history questionnaire (DHQ) or by a brief diet history questionnaire (BDHQ) in Japanese women (*n* = 121; (**a**) DHQ and (**b**) BDHQ) and men (*n* = 121; (**c**) DHQ and (**d**) BDHQ). In each season, a 4-day weighed DR was conducted: November and December 2002 (autumn), February 2003 (winter), May 2003 (spring) and August and September 2003 (summer). For each individual, the mean value of the 16-day data was used. The DHQ and BDHQ used in this analysis were conducted before the period of the DR (November 2002). A regression line was added when it was statistically significant (*p* < 0.05).

**Figure 2:**
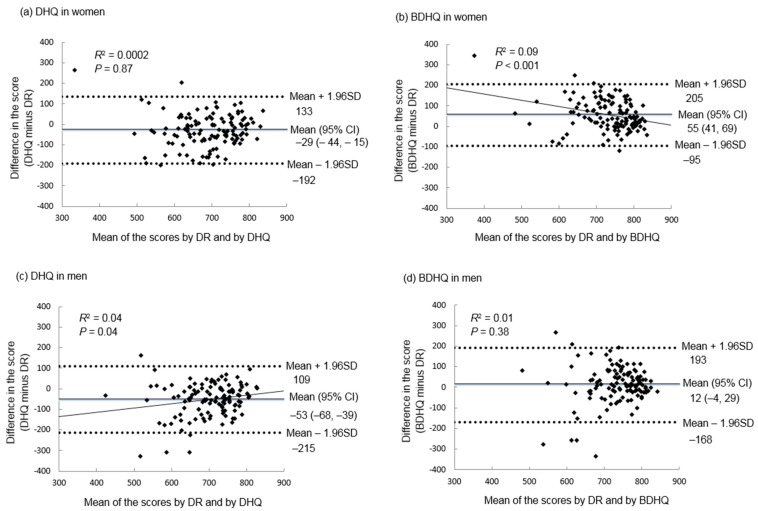
Bland–Altman plots assessing the agreement between the total score of the Nutrient-Rich Food Index 9.3 estimated by a 16-day weighed dietary record (DR) conducted over a year and that estimated by a comprehensive diet history questionnaire (DHQ) or by a brief diet history questionnaire (BDHQ) in Japanese women (*n* = 121; (**a**) DHQ and (**b**) BDHQ) and men (*n* = 121; (**c**) DHQ and (**d**) BDHQ). In each season, a 4-day weighed DR was conducted: November and December 2002 (autumn), February 2003 (winter), May 2003 (spring) and August and September 2003 (summer). For each individual, the mean value of the 16-day data was used. The DHQ and BDHQ used in this analysis were conducted before the period of the DR (November 2002). A regression line was added when it was statistically significant (*p* < 0.05).

Bland–Altman plots were applied for assessing the agreement between the total score of HEI-2015 (Figure 1) and of NRF9.3 (Figure 2) estimated by the DR and that by the DHQ or BDHQ. Except for the HEI-2015 estimated by DHQ and the NRF9.3 estimated by BDHQ in men, the mean difference was significantly different from zero. In all cases, the limit of agreement (mean difference ± 1.96 SD of the difference) was wide, indicating poor agreement. For the DHQ, there was a weak but significant tendency of overestimation for higher levels of the score and underestimation for lower levels of the score, except for the NRF9.3 in women. For the BDHQ, an opposite tendency (i.e., underestimation for higher levels of the score and overestimation for lower levels of the score) was observed only in women.

Associations between the intakes of selected nutrients and the total scores of HEI-2015 and NRF 9.3 (based on DR) are shown in Table 4 and Table S3, respectively. As expected, higher total scores of HEI-2015 and NRF9.3 were associated with favourable profiles of nutrient intake patterns in both sexes, including higher intakes of dietary fibre and key vitamins and minerals. Nevertheless, no association was seen for the intake of sodium (both HEI-2015 and NRF9.3) or added sugars (HEI-2015), mainly because of high and low intake compared with the reference value, respectively.

## 4. Discussion

To our knowledge, this is the first study to investigate the reproducibility and relative validity of the HEI-2015 and NRF9.3 estimated from intake assessed by dietary assessment questionnaires. Furthermore, only very few studies have examined this issue using other diet quality measures. For the Diet Quality Index Revised calculated by a food frequency questionnaire (FFQ), a reproducibility correlation of 0.72 and validity correlation of 0.66 (compared with a 14-day DR) were observed in 127 US men [34]. The correlations between a modified Mediterranean diet score and a Mediterranean-like diet score estimated by an FFQ and those by ≥10 × 24-h dietary recall were 0.48 and 0.62, respectively, in 107 Spanish adults [35]. A correlation of 0.48 was found between the Dutch Healthy Diet index calculated based on an FFQ and that based on 2 × 24-h dietary recall in 121 subjects [36]. In this study of Japanese adults, we found that the intraclass correlations between the two DHQs or two BDHQs completed one year apart ranged from 0.53 to 0.77, depending on sex and dietary assessment questionnaire, showing reasonable reproducibility. We also found that the Pearson correlations for HEI-2015 and NRF9.3 between the DHQ or BDHQ and a 16-day DR (conducted over one year) ranged from 0.37 to 0.61. The percentage of participants who were classified in the same or adjacent quintile of the scores in the DHQ or BDHQ and DR ranged from 61.9% to 75.2%. These results suggest reasonable ranking ability. Additionally, a single administration of our DHQ or BDHQ (to assess dietary habits during the preceding month) seemed to be reflective of overall diet quality over a longer period (i.e., one year) with relative accuracy, seemingly due to relatively small seasonal variation in intake. Thus, our present findings support the future efficacy of both the DHQ and BDHQ for estimating the HEI-2015 and NRF9.3 as measures of overall quality of Japanese diets in future epidemiological studies of the potential health effects of overall diets in Japan.

Nevertheless, we observed poor agreement between both the HEI-2015 and NRF9.3 estimated by the DHQ or BDHQ and by DR. This is consistent with the previous study of diet quality score mentioned above [35] as well as with observation of the DHQ and BDHQ at the food level [19]. Thus, the absolute score of HEI-2015 and NRF9.3 should be interpreted with considerable caution at the individual level.

In this study, the relative validity (particularly ranking ability) was slightly higher, with the DHQ than BDHQ for both the HEI-2015 and NRF9.3, particularly in men. This was not apparent in previous validation studies of the DHQ and BDHQ at the food and nutrient level, in which the DHQ and BDHQ showed a similar ranking ability [19,20]. Although the reason is not precisely known, this present finding may reflect differences in the amount of information available between the DHQ (151 food items; 236 food codes in algorithm; information on portion size) and BDHQ (58 food items; 150 food codes in algorithm; no information on portion size). These features of the BDHQ should act to lessen the variation in dietary intake, which might be of particular importance when a composite measure of dietary intake is calculated.

Despite the lack of any clear difference in the ranking ability of the DHQ and BDHQ at the food and nutrient level between men and women [19,20], we observed that the ranking ability for HEI-2015 and NRF9.3 was somewhat higher in women than men. Again, no precise explanation for this finding is available, but it may be due to the smaller variation in component scores in men than women, which is particularly evident by the presence of a larger number of male participants with the maximum score (e.g., added sugars and saturated fats in HEI-2015 and vitamin D and iron in NRF9.3). If so, the use of a different scoring system and different cut-off points to increase (or maximize) the distribution would apparently improve the ranking ability of both diet quality measures, which might be particularly relevant in cases such as diet and disease investigations. Nevertheless, this ceiling in the HEI-2015 and NRF9.3 should not be considered a limitation, because it would be inappropriate to give higher scores to diets that exceed the standard given that consuming more than the recommended amount of any food or nutrient might not confer any advantage [37].

The present finding might also be interpreted to be evidence that a dietary assessment questionnaire with reasonable validity at the food and nutrient level can nevertheless provide a valid estimate of a composite measure of overall diet quality. If so, this study, based on the DHQ and BDHQ, would facilitate, for example, prospective cohort studies on health effects of the overall quality of Japanese diets as assessed by HEI-2015 or NRF9.3, even using different dietary assessment questionnaires. Furthermore, it may also be of interest to conduct an international comparison (particularly in Asian countries) of diet quality as assessed by HEI-2015 or NRF9.3 using different dietary assessment methods, albeit that this might also require the development of suitable calibration methods.

Although higher total HEI-2015 and NRF9.3 scores were associated with favourable dietary intake pattern profiles, scores were not associated with sodium or added sugar intake. This is mainly because, on average, sodium intake was too high while added sugar intake was low compared with the reference value. It may be prudent to use different reference values which are more reflective of current intake levels, although more research is needed to decide such values.

One limitation of this study is that we used a DR as a reference method even though DRs are also susceptible to measurement error due to erroneous recording and potential changes in eating behaviour [38]. Particularly, underreporting of energy intake is common in any dietary surveys [38]; thus, significant difference in mean energy intake between the DHQ or BDHQ and DR observed in this study may not necessarily cause question on the validity of the DHQ or BDHQ. Nevertheless, the potential influence of energy intake underreporting should be minimized in this study because the calculation of HEI-2015 and NRF9.3 was based on energy-adjusted estimates of dietary intakes. Furthermore, errors in a DR are thought to have lesser correlation with errors in a dietary assessment questionnaire than errors in a 24-h dietary recall or other instruments that rely on memory [39]. In any case, a validity investigation using biological markers (blood and urine) would strengthen the usefulness of the DHQ and BDHQ in estimating HEI-2015 and NRF9.3. In this context, the comparison of the HEI-2015 and NRF9.3 derived from the DHQ or BDHQ between healthy and non-healthy groups would also be valuable. A second limitation is that our participants were not a representative sample of general Japanese but rather volunteers, and thus possibly health conscious. Moreover, the survey areas were not equally distributed over the country but located mostly in the western parts of Japan. Thus, the generalizability of our present results may be limited.

## 5. Conclusions

These data indicate the reasonable reproducibility and ranking ability of the DHQ and BDHQ for assessing the HEI-2015 and NRF9.3, although the agreement between the DR and DHQ or BDHQ was poor. This finding supports their usefulness in future epidemiological research on the overall effects of Japanese diets on various health outcomes. One caveat is that this promising finding should not be interpreted to mean that these diet quality measures are consistently useful in disease outcome prediction. Accordingly, an important future step in evaluating the utility of the HEI-2015 and NRF9.3 in dietary assessment is to investigate whether they reliably predict disease risk.

## Figures and Tables

**Table 1:** Basic characteristics of study participants ^a^.

Variable	Women (*n* = 121)	Men (*n* = 121)
Age (years)	49.6 ± 11.3	52.4 ± 12.2
Body height (m)	1.55 ± 0.06	1.67 ± 0.07
Body weight (m)	53.3 ± 7.1	66.4 ± 10.4
Body mass index (kg/m^2^)	22.3 ± 2.8	23.6 ± 2.9
Energy intake (kJ/day)		
16-day DR ^b^	7697 ± 1203	9888 ± 1753
DHQ ^c^	8177 ± 1825 *	9656 ± 2241
BDHQ ^d^	7119 ± 1731 *	9050 ± 2280 *

BDHQ, brief diet history questionnaire; DHQ, diet history questionnaire; DR, dietary record. ^a^ Values are means ± standard deviations. ^b^ In each season, a 4-day weighed DR was conducted: November and December 2002 (autumn), February 2003 (winter), May 2003 (spring) and August and September 2003 (summer). For each individual, the mean value of the 16-day data was used. ^c^ Based on the DHQ conducted prior to the DR period (November 2002). ^d^ Based on the BDHQ conducted prior to the DR period (November 2002). Mean energy intake derived from the DHQ or BDHQ was compared with that derived from the 16-day DR in each sex, based on paired *t*-test: * *p* < 0.05.

**Table 2:** Component and total scores of the Healthy Eating Index-2015 estimated from a 16-day weighed dietary record (DR) conducted over a year and a comprehensive diet history questionnaire (DHQ) and a brief diet history questionnaire (BDHQ) completed before and after the period of DR in Japanese women and men ^a^.

	DR ^b^	DHQ1 ^c^	DHQ2 ^d^	BDHQ1 ^c^	BDHQ2 ^d^	Intraclass Correlation (Reproducibility) ^e^		Pearson Correlation with DR (Relative Validity)		% of Participants with Maximum Score ^f^		
						DHQ1	BDHQ1	DHQ1	BDHQ1	DR	DHQ1	BDHQ1
**Women (*n* = 121)**												
Total fruits	2.6 ± 1.4	3.0 ± 1.5 ***	2.9 ± 1.5	3.0 ± 1.5 **	3.0 ± 1.6	0.61	0.69	0.54	0.50	5.0	19.0	19.8
Whole fruits	3.8 ± 1.6	4.0 ± 1.5	4.0 ± 1.5	4.0 ± 1.4	4.0 ± 1.4	0.65	0.73	0.54	0.54	48.8	57.9	54.6
Total vegetables	4.8 ± 0.5	4.5 ± 0.9 ***	4.4 ± 0.9	4.8 ± 0.6	4.8 ± 0.5	0.55	0.69	0.46	0.63	85.1	58.7	89.3
Greens and beans	3.8 ± 1.3	3.1 ± 1.5 ***	3.2 ± 1.5	4.4 ± 1.2 ***	4.2 ± 1.2	0.43	0.27	0.48	0.30	33.9	21.5	66.9
Whole grains	0.7 ± 1.4	1.1 ± 1.9 *	1.2 ± 2.1	0.5 ± 0.6	0.7 ± 1.0 *	0.45	0.30	0.42	0.18	0	1.7	0
Dairy	2.9 ± 1.5	3.3 ± 2.1 *	3.0 ± 1.8	2.9 ± 1.8	2.7 ± 1.7	0.47	0.39	0.49	0.40	0	2.5	0
Total protein foods	4.9 ± 0.2	4.7 ± 0.6 ***	4.8 ± 0.5	4.9 ± 0.4	4.9 ± 0.3	0.55	0.21	0.24	0.11	85.1	63.6	87.6
Seafood and plant proteins	5.0 ± 0.2	4.9 ± 0.4 *	5.0 ± 0.2	5.0 ± 0.2	5.0 ± 0.3	0.18	0.76	0.13	0.78	97.5	91.7	96.7
Fatty acids	6.4 ± 1.9	7.2 ± 2.4 ***	7.6 ± 2.2	7.5 ± 2.3 ***	7.9 ± 2.1*	0.57	0.59	0.45	0.37	4.1	19.8	26.5
Refined grains	1.2 ± 1.7	1.9 ± 2.5 **	2.0 ± 2.6	1.3 ± 2.1	1.1 ± 1.7	0.49	0.25	0.47	0.37	0	2.5	0
Sodium	0.7 ± 1.7	1.0 ± 1.9	1.4 ± 2.5	0.6 ± 1.3	0.7 ± 1.7	0.38	0.56	0.40	0.38	0	0	0
Added sugars	9.5 ± 1.0	9.4 ± 0.9	9.3 ± 0.9	9.8 ± 0.6 **	9.8 ± 0.6	0.52	0.75	0.51	0.17	55.4	45.5	80.2
Saturated fats	9.1 ± 1.2	9.3 ± 1.2	9.5 ± 1.1	9.7 ± 0.7 ***	9.8 ± 0.6	0.48	0.03	0.32	0.16	44.6	54.6	71.9
Total score ^g^	55.4 ± 6.0	57.3 ± 7.2 **	58.0 ± 7.1	58.3 ± 4.7 ***	58.6 ± 5.1	0.58	0.57	0.57	0.52	0	0	0
**Men (*n* = 121)**												
Total fruits	1.8 ± 1.3	2.1 ± 1.5 ***	2.1 ± 1.5	2.3 ± 1.6 ***	2.3 ± 1.6	0.72	0.68	0.68	0.57	5.0	6.6	9.9
Whole fruits	2.9 ± 1.7	3.1 ± 1.8 *	3.1 ± 1.8	3.2 ± 1.7 *	3.2 ± 1.7	0.75	0.68	0.65	0.52	20.7	33.9	35.5
Total vegetables	4.8 ± 0.5	3.7 ± 1.2 ***	3.7 ± 1.2	4.5 ± 1.0 ***	4.6 ± 0.7	0.49	0.47	0.36	0.43	76.9	28.9	70.3
Greens and beans	3.3 ± 1.3	2.3 ± 1.4 ***	2.5 ± 1.4	3.9 ± 1.4 ***	4.0 ± 1.3	0.44	0.26	0.40	0.25	20.7	8.3	50.4
Whole grains	0.7 ± 1.6	1.0 ± 2.0	1.2 ± 2.2	0.7 ± 0.9	0.7 ± 0.9	0.53	0.56	0.48	0.32	0	3.3	0
Dairy	1.9 ± 1.4	2.0 ± 1.7	2.1 ± 1.7	1.9 ± 1.7	2.2 ± 1.7	0.62	0.51	0.61	0.46	0	0.8	0
Total protein foods	4.9 ± 0.2	4.6 ± 0.7 ***	4.6 ± 0.7	4.7 ± 0.6 ***	4.8 ± 0.5	0.21	0.41	−0.02	0.02	83.5	56.2	70.3
Seafood and plant proteins	5.0 ± 0.2	4.9 ± 0.4 **	4.8 ± 0.7	4.9 ± 0.5	5.0 ± 0.2	0.09	0.32	0.62	−0.02	98.4	91.7	93.4
Fatty acids	7.2 ± 2.0	8.0 ± 2.1 ***	8.2 ± 2.0	8.1 ± 2.2 ***	8.2 ± 2.3	0.51	0.48	0.44	0.29	8.3	33.9	40.5
Refined grains	1.2 ± 2.0	1.7 ± 2.7 **	1.8 ± 2.7	1.5 ± 2.3	1.6 ± 2.2	0.51	0.53	0.54	0.58	0	4.1	1.7
Sodium	1.1 ± 1.9	1.9 ± 2.5 **	2.0 ± 2.7	1.0 ± 2.1	1.2 ± 2.1	0.50	0.52	0.27	0.42	0	0	0
Added sugars	9.8 ± 0.8	9.6 ± 0.9 *	9.6 ± 1.0	9.8 ± 0.7	9.8 ± 0.8	0.64	0.34	0.58	0.60	79.3	65.3	82.6
Saturated fats	9.7 ± 0.9	9.8 ± 0.7 **	9.8 ± 1.0	9.9 ± 0.5 *	9.9 ± 0.7	0.75	0.23	0.65	0.22	74.4	81.0	90.1
Total score ^g^	54.3 ± 5.5	54.8 ± 7.2	55.3 ± 7.6	56.5 ± 5.8 ***	57.3 ± 5.0	0.57	0.53	0.51	0.43	0	0	0

^a^ For each of the component and total scores of Healthy Eating Index-2015, a higher score indicates a higher diet quality. Values are means ± standard deviations unless otherwise indicated. ^b^ In each season, a 4-day weighed DR was conducted: November and December 2002 (autumn), February 2003 (winter), May 2003 (spring) and August and September 2003 (summer). For each individual, the mean value of the 16-day data was used. ^c^ Based on the DHQ or BDHQ conducted before the DR period (November 2002). Mean values derived from the DHQ1 or BDHQ1 were compared with those derived from the DR based on paired *t*-test: * *p* < 0.05; ** *p* < 0.01; *** *p* < 0.001. ^d^ *n* = 120. Based on the DHQ or BDHQ conducted after the DR period (November 2003). Mean values derived from the DHQ2 or BDHQ2 were compared with those derived from the DHQ1 or BDHQ1 based on paired *t*-test: * *p* <0.05. ^e^ *n* = 120. ^f^ The maximum score is as follows: 5 for total fruits, whole fruits, total vegetables, greens and beans, total protein foods and seafood and plant proteins; 10 for whole grains, dairy, fatty acids, refined grains, sodium, added sugars and saturated fats; 100 for total score. The percentages of participants with maximum score based on DHQ2 and BDHQ2 were similar to those based on DHQ1 and BDHQ1, respectively. ^g^ Calculated as the sum of all component scores.

**Table 3:** Component and total scores of the Nutrient-Rich Food Index 9.3 estimated from a 16-day weighed dietary record (DR) conducted over a year and a comprehensive diet history questionnaire (DHQ) and a brief diet history questionnaire (BDHQ) completed before and after DR in Japanese women and men ^a^.

	DR ^b^	DHQ1 ^c^	DHQ2 ^d^	BDHQ1 ^c^	BDHQ2 ^d^	Intraclass Correlation (Reproducibility) ^e^		Pearson Correlation with DR (Relative Validity)		% of Participants with Maximum score ^f^		
						DHQ1	BDHQ1	DHQ1	BDHQ1	DR	DHQ1	BDHQ1
**Women (*n* = 121)**												
Protein	100 ± 0	100 ± 1	100 ± 1	100 ± 0	100 ± 0	0.49	NA ^g^	NA ^g^	NA ^g^	100	97.5	100
Dietary fibre	83 ± 16	79 ± 17**	78 ± 17	83 ± 16	80 ± 16*	0.66	0.64	0.69	0.61	21.5	19.8	23.1
Vitamin A	79 ± 18	79 ± 20	81 ± 20	93 ± 13 ***	93 ± 12	0.56	0.72	0.41	0.38	28.9	31.4	70.3
Vitamin C	93 ± 15	92 ± 15	90 ± 17	97 ± 12 ***	96 ± 12	0.48	0.68	0.51	0.62	69.4	70.3	89.3
Vitamin D	96 ± 10	94 ± 14	96 ± 12	99 ± 7 **	99 ± 5	0.57	0.01	0.24	0.06	76.0	77.7	93.4
Calcium	86 ± 15	83 ± 17	82 ± 17	91 ± 13 ***	88 ± 15*	0.65	0.60	0.48	0.53	34.7	30.6	47.9
Iron	88 ± 15	84 ± 18 ***	84 ± 17	91 ± 13 ***	91 ± 14	0.85	0.79	0.88	0.82	54.6	43.8	56.2
Potassium	96 ± 7	93 ± 11 **	92 ± 12	98 ± 7 ***	97 ± 8	0.65	0.60	0.48	0.57	64.5	47.9	86.8
Magnesium	95 ± 9	89 ± 12 ***	89 ± 11	95 ± 8	94 ± 9	0.60	0.65	0.52	0.55	63.6	28.9	57.0
Added sugars	35 ± 45	43 ± 43	45 ± 43	17 ± 33 ***	17 ± 33	0.57	0.76	0.53	0.31	34.7	20.7	58.7
Saturated fats	19 ± 17	16 ± 18	13 ± 17*	9 ± 13 ***	7 ± 12	0.51	0.16	0.44	0.30	23.1	31.4	50.4
Sodium	57 ± 25	61 ± 35	53 ± 32**	62 ± 28	59 ± 29	0.51	0.45	0.35	0.37	1.7	3.3	1.7
Total score ^h^	704 ± 93	675 ± 92 ***	682 ± 88	759 ± 73 ***	757 ± 75	0.74	0.77	0.61	0.61	0	0	0
**Men (*n* = 121)**												
Protein	100 ± 0	100 ± 2	100 ± 3	100 ± 1	100 ± 0	−0.02	−0.01	NA ^g^	NA ^g^	100	95.9	97.5
Dietary fibre	81 ± 15	72 ± 18 ***	73 ± 19	79 ± 18	77 ± 18	0.58	0.58	0.64	0.55	24.0	10.7	22.3
Vitamin A	73 ± 21	66 ± 25 **	68 ± 23	85 ± 20 ***	87 ± 17	0.49	0.43	0.34	0.19	26.5	23.1	53.7
Vitamin C	95 ± 11	89 ± 18 ***	89 ± 18	96 ± 14	96 ± 12	0.23	0.31	0.38	0.34	72.7	55.4	86.0
Vitamin D	99 ± 4	98 ± 8	98 ± 9	98 ± 10	99 ± 5	0.28	0.33	0.40	0.30	90.9	88.4	95.9
Calcium	85 ± 14	77 ± 18 ***	78 ± 18	89 ± 16 *	90 ± 14	0.55	0.51	0.53	0.54	29.8	19.8	50.4
Iron	100 ± 2	96 ± 7 ***	96 ± 9	98 ± 6 *	99 ± 4	0.33	0.25	0.03	0.26	91.7	70.3	89.3
Potassium	96 ± 8	87 ± 13 ***	86 ± 14	95 ± 10	95 ± 9	0.53	0.53	0.39	0.40	61.2	28.9	70.3
Magnesium	93 ± 9	84 ± 12 ***	84 ± 13	92 ± 11	91 ± 10	0.55	0.57	0.47	0.46	47.9	18.2	38.0
Added sugars	19 ± 38	29 ± 44 *	31 ± 46	15 ± 33	14 ± 40	0.65	0.39	0.52	0.51	53.7	44.6	65.3
Saturated fats	10 ± 14	6 ± 12 ***	6 ± 15	4 ± 10 ***	3 ± 11	0.66	0.26	0.63	0.26	47.9	64.5	78.5
Sodium	65 ± 25	60 ± 35	60 ± 36	74 ± 35 **	72 ± 36	0.44	0.53	0.38	0.41	0	4.1	0.8
Total score ^h^	728 ± 77	674 ± 91 ***	673 ± 88	740 ± 83	745 ± 75	0.61	0.56	0.55	0.37	0	0	0

NA, not applicable. ^a^ In Nutrient-Rich Food Index 9.3, a higher score indicates a higher diet quality, except for added sugars, saturated fats and sodium components, for which a higher score indicates an unfavorable dietary intake (i.e., higher intakes of added sugars, saturated fats and sodium). Values are means ± standard deviations unless otherwise indicated. ^b^ In each season, a 4-day weighed DR was conducted: November and December 2002 (autumn), February 2003 (winter), May 2003 (spring) and August and September 2003 (summer). For each individual, the mean value of the 16-day data was used. ^c^ Based on the DHQ or BDHQ conducted before the DR period (November 2002). Mean values derived from the DHQ1 or BDHQ1 were compared with those derived from the DR based on paired *t*-test: * *p* < 0.05; ** *p* < 0.01; *** *p* < 0.001. ^d^ *n* = 120. Based on the DHQ or BDHQ conducted after the DR period (November 2003). Mean values derived from the DHQ2 or BDHQ2 were compared with those derived from the DHQ1 or BDHQ1 based on paired *t*-test: * *p* < 0.05; ** *p* < 0.01. ^e^ *n* = 120. ^f^ The maximum (most favorable) score is as follows: 100 for protein, dietary fiber, vitamins A, C and D, calcium, iron, potassium and magnesium; 0 for added sugars, saturated fats and sodium; 900 for total score. The percentages of participants with maximum score based on DHQ2 and BDHQ2 were similar to those based on DHQ1 and BDHQ1, respectively. ^g^ Not calculated because all participants had the maximum score of 100 at least in one dietary assessment. ^h^ Calculated as the sum of scores for nine nutrients to encourage (i.e., protein, dietary fibre, vitamins A, C, and D, calcium, iron, potassium and magnesium) minus the sum of scores for three nutrients to limit (i.e., added sugar, saturated fats and sodium).

**Table 4:** Dietary intake according to tertile category of the HEI-2015 total score estimated from a 16-day weighed dietary record in Japanese women and men ^a^.

	Women				Men			
	T1 (*n* = 40)	T2 (*n* = 41)	T3 (*n* =40)	*p* for trend ^b^	T1 (*n* = 40)	T2 (*n* = 41)	T3 (*n* = 40)	*p* for trend ^b^
HEI-2015 total score ^c^	48.7 ± 3.7	55.9 ± 1.5	61.6 ± 3.1		48.6 ± 1.9	53.7 ± 1.3	60.5 ± 3.5	
HEI-2015 total score (median)	49.6	55.9	60.3		49.1	53.8	59.7	
HEI-2015 score components								
Total fruits (cup Eq/4184 kJ)	0.2 ± 0.2	0.4 ± 0.2	0.6 ± 0.2	<0.0001	0.1 ± 0.1	0.2 ± 0.2	0.5 ± 0.3	<0.0001
Whole fruits (cup Eq/4184 kJ)	0.2 ± 0.2	0.3 ± 0.6	0.6 ± 0.2	<0.0001	0.1 ± 0.1	0.2 ± 0.2	0.5 ± 0.3	<0.0001
Total vegetables (cup Eq/4184 kJ)	1.3 ± 0.5	1.7 ± 0.4	1.9 ± 0.5	<0.0001	1.1 ± 0.3	1.3 ± 0.3	1.7 ± 0.3	<0.0001
Greens and beans (cup Eq/4184 kJ)	0.1 ± 0.1	0.2 ± 0.1	0.2 ± 0.1	<0.0001	0.1 ± 0.0	0.1 ± 0.0	0.2 ± 0.1	<0.0001
Whole grains (ounce Eq/4184 kJ)	0.0 ± 0.1	0.1 ± 0.1	0.2 ± 0.3	0.0001	0.0 ± 0.1	0.1 ± 0.1	0.2 ± 0.4	0.003
Dairy (cup Eq/4184 kJ)	0.3 ± 0.2	0.4 ± 0.2	0.4 ± 0.2	0.04	0.2 ± 0.2	0.2 ± 0.2	0.3 ± 0.2	0.0003
Total protein foods (ounce Eq/4184 kJ)	2.9 ± 0.5	3.2 ± 0.6	3.4 ± 0.6	<0.0001	3.0 ± 0.5	3.1 ± 0.7	3.3 ± 0.7	0.006
Seafood and plant proteins (cup Eq/4184 kJ)	1.5 ± 0.5	2.1 ± 0.6	2.3 ± 0.5	<0.0001	1.5 ± 0.5	1.9 ± 0.7	2.2 ± 0.6	<0.0001
Fatty acids ^d^	1.9 ± 0.2	2.1 ± 0.2	2.1 ± 0.3	0.0003	2.1 ± 0.2	2.2 ± 0.3	2.2 ± 0.3	0.33
Refined grains (ounce Eq/4184 kJ)	4.5 ± 0.7	4.4 ± 0.4	3.8 ± 0.7	<0.0001	4.5 ± 0.7	4.4 ± 0.7	3.9 ± 0.8	0.0004
Sodium (mg/4184 kJ)	2177 ± 361	2196 ± 361	2289 ± 383	0.18	2046 ± 312	2061 ± 308	2105 ± 378	0.43
Added sugars (% of energy)	6.8 ± 3.1	6.0 ± 2.2	6.3 ± 2.5	0.45	5.8 ± 3.4	4.4 ± 2.0	5.1 ± 1.9	0.21
Saturated fats (% of energy)	9.2 ± 1.4	7.8 ± 1.2	7.6 ± 1.3	<0.0001	7.7 ± 1.6	7.0 ± 1.3	6.9 ± 1.3	0.01
Selected nutrients								
Protein (% of energy)	14.2 ± 1.3	15.1 ± 1.5	15.9 ± 1.7	<0.0001	13.7 ± 1.3	14.1 ± 1.5	15.2 ± 1.8	<0.0001
Fat (% of energy)	30.0 ± 3.6	27.3 ± 3.4	27.0 ± 3.3	0.0002	26.9 ± 3.9	25.1 ± 3.6	25.0 ± 3.8	0.03
MUFA (% of energy)	11.1 ± 1.8	9.9 ± 1.6	9.6 ± 1.5	0.0001	10.2 ± 1.8	9.3 ± 1.6	9.1 ± 1.8	0.003
PUFA (% of energy)	6.2 ± 0.8	6.3 ± 0.7	6.3 ± 0.8	0.54	5.8 ± 0.7	5.8 ± 0.9	5.8 ± 1.0	0.99
Carbohydrate (% of energy)	52.8 ± 4.3	55.5 ± 3.7	55.4 ± 4.9	0.009	52.9 ± 5.6	52.0 ± 6.1	53.9 ± 6.2	0.45
Alcohol (% of energy)	1.8 ± 2.8	1.1 ± 1.5	1.3 ± 2.3	0.38	4.7 ± 4.1	7.3 ± 6.8	5.0 ± 5.8	0.83
Dietary fibre (g/4184 kJ)	6.4 ± 1.5	7.9 ± 1.2	9.6 ± 1.9	<0.0001	5.4 ± 0.9	6.2 ± 1.2	8.4 ± 1.4	<0.0001
Vitamin A (μg RAE/4184 kJ)	305 ± 201	362 ± 236	407 ± 178	0.03	284 ± 218	295 ± 154	383 ± 284	0.0501
Vitamin D (μg/4184 kJ)	3.4 ± 1.3	4.6 ± 2.1	4.8 ± 1.6	0.0005	3.3 ± 1.4	4.2 ± 1.8	4.9 ± 1.7	<0.0001
Vitamin E (mg/4184 kJ)	3.8 ± 0.7	4.0 ± 0.5	4.4 ± 0.6	<0.0001	3.3 ± 0.4	3.5 ± 0.5	4.0 ± 0.6	<0.0001
Vitamin K (μg/4184 kJ)	94 ± 28	133 ± 37	163 ± 50	<0.0001	81 ± 21	102 ± 31	135 ± 32	<0.0001
Thiamine (mg/4184 kJ)	0.5 ± 0.1	0.5 ± 0.1	0.5 ± 0.1	<0.0001	0.4 ± 0.1	0.4 ± 0.1	0.5 ± 0.1	0.0001
Riboflavin (mg/4184 kJ)	0.6 ± 0.1	0.7 ± 0.1	0.8 ± 0.1	<0.0001	0.6 ± 0.1	0.6 ± 0.1	0.7 ± 0.1	<0.0001
Niacin (mg/4184 kJ)	8.8 ± 1.2	9.1 ± 1.6	10.2 ±2.4	0.0004	9.0 ± 1.6	9.4 ± 1.7	9.8 ± 2.2	0.04
Vitamin B-6 (mg/4184 kJ)	0.6 ± 0.1	0.7 ± 0.1	0.8 ± 0.1	<0.0001	0.6 ± 0.1	0.6 ± 0.1	0.7 ± 0.1	<0.0001
Vitamin B-12 (μg/4184 kJ)	3.6 ± 1.3	4.6 ± 1.7	4.6 ± 1.5	0.004	3.6 ± 1.5	4.2 ± 1.7	4.7 ± 1.7	0.002
Folate (μg/4184 kJ)	156 ± 41	198 ± 41	230 ± 41	<0.0001	141 ± 32	167 ± 44	205 ± 43	<0.0001
Vitamin C (mg/4184 kJ)	44.5 ± 16.3	65.4 ± 19.4	76.5 ± 15.2	<0.0001	36.8 ± 9.6	47.5 ± 12.2	70.1 ± 18.3	<0.0001
Potassium (mg/4184 kJ)	1242 ± 211	1445 ± 196	1695 ± 236	<0.0001	1099 ± 138	1209 ± 176	1502 ± 218	<0.0001
Calcium (mg/4184 kJ)	268 ± 62	302 ± 64	348 ± 75	<0.0001	213 ± 55	229 ± 53	305 ± 72	<0.0001
Magnesium (mg/4184 kJ)	135 ± 25	158 ± 22	181 ± 25	<0.0001	125 ± 18	138 ± 20	165 ± 24	<0.0001
Iron (mg/4184 kJ)	3.8 ± 0.6	4.4 ± 0.7	5.0 ± 0.8	<0.0001	3.5 ± 0.6	3.8 ± 0.6	4.5 ± 0.8	<0.0001
Zinc (mg/4184 kJ)	4.3 ± 0.4	4.4 ± 0.4	4.6 ± 0.4	0.0006	4.2 ± 0.4	4.1 ± 0.5	4.5 ± 0.5	0.01
Energy (kJ/d)	7479 ± 1441	7890 ± 1081	7718 ± 1044	0.38	9896 ± 1805	9988 ± 1518	9775 ± 1951	0.76

Eq, equivalents; HEI-2015, Healthy Eating Index-2015; RAE, retinol activity equivalent; T, tertile. ^a^ In each season, a 4-day weighed dietary record was conducted: November and December 2002 (autumn), February 2003 (winter), May 2003 (spring) and August and September 2003 (summer). For each individual, the mean value of the 16-day data was used. Values are means ± standard deviations unless otherwise indicated. ^b^ Calculated based on general linear models. ^c^ A higher score indicates a higher diet quality. ^d^ Ratio of the sum of polyunsaturated and monounsaturated fatty acids (in g) to saturated fatty acids (in g).

## Supplementary Materials

The following are available online at https://www.mdpi.com/2072-6643/11/10/2540/s1, Table S1: Age- and sex-specific reference daily values used for the calculation of NRF9.3, Table S2: HEI-2015 and NRF9.3 total scores estimated from a 4-day weighed dietary record (DR), a comprehensive diet history questionnaire (DHQ) and a brief diet history questionnaire (BDHQ) conducted in each season over one year and intraclass correlation in Japanese women and men, Table S3: Dietary intake according to tertile category of the NRF9.3 total score estimated from a 16-day weighed dietary record in Japanese women and men, Figure S1: Relationship between the total score of the Healthy Eating Index-2015 estimated by a 16-day weighed dietary record (DR) conducted over a year and that estimated by a comprehensive diet history questionnaire (DHQ) or by a brief diet history questionnaire (BDHQ) in Japanese women (*n* 121) and men (*n* 121). In each season, a 4-day weighed DR was conducted: November and December 2002 (autumn), February 2003 (winter), May 2003 (spring) and August and September 2003 (summer). For each individual, the mean value of the 16-day data was used. The DHQ and BDHQ used in this analysis were conducted before the period of the DR (November 2002), Figure S2: Relationship between the total score of the Nutrient-Rich Food Index 9.3 estimated by a 16-day weighed dietary record (DR) conducted over a year and that estimated by a comprehensive diet history questionnaire (DHQ) or by a brief diet history questionnaire (BDHQ) in Japanese women (*n* 121) and men (*n* 121). In each season, a 4-day weighed DR was conducted: November and December 2002 (autumn), February 2003 (winter), May 2003 (spring) and August and September 2003 (summer). For each individual, the mean value of the 16-day data was used. The DHQ and BDHQ used in this analysis were conducted before the period of the DR (November 2002).
