# Supplementary material for: Reproducibility and Relative Validity of the Healthy Eating Index-2015 and Nutrient-Rich Food Index 9.3 Estimated by Comprehensive and Brief Diet History Questionnaires in Japanese Adults
Source: Nutrients. 2019 Oct 21;11(10):2540. doi: 10.3390/nu11102540 (PMC6836176; doi:10.3390/nu11102540)
Supplement: Supplementary file 1 [file nutrients-11-02540-s001.pdf]

Table S1. Age- and sex-specific reference daily values used for the calculation of NRF9.3 [a]

	30-49 years		50-69 years		≥70 years	
	Women	Men	Women	Men	Women	Men
Energy (kcal)[b]	2000	2650	1900	2450	1750	2200
Energy (kJ)[c]	8368	11088	7950	10251	7322	9205
Qualifying nutrients						
Protein (g)[c]	50	60	50	60	50	60
Dietary fibre (g)[d]	18	20	18	20	17	19
Vitamin A (μg RAE)[c]	700	900	700	850	650	800
Vitamin C (mg)[c]	100	100	100	100	100	100
Vitamin D (μg)[e]	5.5	5.5	5.5	5.5	5.5	5.5
Calcium (mg)[c]	650	650	650	700	650	700
Iron (mg)[c]	10.5	7.5	6.5	7.5	6	7
Potassium (mg)[d]	2600	3000	2600	3000	2600	3000
Magnesium (mg)[c]	290	370	290	350	270	320
Disqualifying nutrients						
Added sugars (g)[f]	25	33.1	23.8	30.6	21.9	27.5
Saturated fats (g)[g]	15.6	20.6	14.8	19.1	13.6	17.1
Sodium (g NaCl equivalent)[dh]	7	8	7	8	7	8

NRF9.3, Nutrient-Rich Food Index 9.3; RAE, retinol activity equivalent. [a] Values were derived from the Dietary Reference Intakes for Japanese, 2015, except for added sugars (as shown below). [b] Estimated Energy Requirement for moderate level of physical activity. [c] Recommended Dietary Allowance. [d] Tentative dietary goal for preventing lifestyle-related diseases (DG). [e] Adequate Intake. [f] Determined based on the World Health Organization's conditional recommendation (5% of energy). [g] Determined based on the DG value (7% of energy). [h] 7 g NaCl equivalent = 2756 mg sodium; 8 g NaCl equivalent = 3150 mg sodium.

Table S2. HEI-2015 and NRF9.3 total scores estimated from a 4-day weighed dietary record (DR), a comprehensive diet history questionnaire (DHQ) and a brief diet history questionnaire (BDHQ) conducted in each season over one year and intraclass correlation in Japanese women and men [a]

	Autumn [b]		Winter [c]		Spring [d]		Summer [e]		Intraclass
	Mean	SD	Mean	SD	Mean	SD	Mean	SD	correlation
Women (n 121)									
HEI-2015									
DR [f]	56.0	6.6	54.1	7.0	52.4	7.4	54.5	7.4	0.51
DHQ	57.3	7.2	55.6	6.7	54.6	6.8	56.1	6.8	0.60
BDHQ	58.3	4.7	57.3	4.5	56.4	5.2	55.5	5.8	0.51
NRF9.3									
DR [f]	698	100	680	102	656	129	673	106	0.62
DHQ	675	92	660	94	643	107	654	99	0.68
BDHQ	759	73	748	73	735	80	715	88	0.59
Men (n 121)									
HEI-2015									
DR [f]	55.2	6.9	53.5	6.9	51.6	6.3	53.4	6.3	0.39
DHQ	54.8	7.2	54.0	6.9	52.7	6.7	53.9	7.2	0.61
BDHQ	56.5	5.8	55.6	5.2	55.1	5.4	54.1	5.0	0.47
NRF9.3									
DR [f]	719	91	706	86	685	96	694	92	0.59
DHQ	674	91	658	89	650	88	660	99	0.55
BDHQ	740	83	733	69	726	83	711	85	0.53

HEI-2015, Healthy Eating Index-2015; NRF9.3, Nutrient-Rich Food Index 9.3. [a] For both HEI-2015 and NRF9.3, a higher score indicates a higher diet quality. [b] November and December 2002 for DR and November for DHQ and BDHQ. [c] February 2003. [d] May 2003. [e] August and September 2003. [f] For each individual, the mean value of the 4-day data was used.

Table S3. Dietary intake according to tertile category of the NRF9.3 total score estimated from a 16-day weighed dietary record in Japanese women and men [a].

	Women				Men			
	T1 (n =40)	T2 (n = 41)	T3 (n =40)	P for trend [b]	T1 (n =40)	T2 (n = 41)	T3 (n =40)	P for trend [b]
NRF9.3 total score [c]	603 ± 83	718 ± 27	790 ± 22	---	642 ± 67	740 ± 19	800 ± 24	---
NRF9.3 total score (median)	625	724	792	---	671	739	796	---
NRF9.3 score components								
Protein (% of energy)	14.0 ± 1.3	15.1 ± 1.4	16.2 ± 1.5	<0.0001	13.5 ± 1.5	14.4 ± 1.4	15.1 ± 1.7	<0.0001
Dietary fibre (g/4184 kJ)	6.3 ± 1.4	8.4 ± 1.7	9.2 ± 1.7	<0.0001	5.3 ± 1.0	6.5 ± 1.4	8.1 ± 1.4	<0.0001
Vitamin A (µg RAE/4184 kJ)	284 ± 154	351 ± 212	441 ± 228	0.001	225 ± 122	331 ± 196	405 ± 295	0.0003
Vitamin C (mg/4184 kJ)	45.4 ± 16.6	63.1 ± 15.6	78.0 ± 18.8	<0.0001	37.0 ± 11.3	50.5 ± 15.0	66.8 ± 19.0	<0.0001
Vitamin D (µg/4184 kJ)	3.2 ± 1.1	4.2 ± 1.6	5.3 ± 1.9	<0.0001	3.3 ± 1.3	4.2 ± 1.8	4.8 ± 1.8	<0.0001
Calcium (mg/4184 kJ)	256 ± 51	311 ± 73	352 ± 65	<0.0001	203 ± 51	246 ± 71	298 ± 61	<0.0001
Iron (mg/4184 kJ)	3.7 ± 0.6	4.4 ± 0.6	5.0 ± 0.7	<0.0001	3.4 ± 0.5	3.9 ± 0.6	4.4 ± 0.8	<0.0001
Potassium (mg/4184 kJ)	1220 ± 206	1494 ± 241	1666 ± 197	<0.0001	1079 ± 164	1258 ± 199	1472 ± 202	<0.0001
Magnesium (mg/4184 kJ)	132 ± 24	163 ± 24	179 ± 22	<0.0001	123 ± 18	143 ± 23	162 ± 22	<0.0001
Added sugars (% of energy)	7.5 ± 3.2	6.7 ± 2.2	4.8 ± 1.6	<0.0001	6.3 ± 3.4	4.6 ± 1.9	4.4 ± 1.6	0.0006
Saturated fats (% of energy)	9.2 ± 1.3	7.7 ± 1.3	7.7 ± 1.2	<0.0001	7.7 ± 1.6	7.2 ± 1.5	6.8 ± 1.1	0.003
Sodium (mg/4184 kJ)	2098 ± 372	2327 ± 369	2234 ± 337	0.10	2030 ± 346	2127 ± 335	2053 ± 315	0.76
Selected nutrients								
Fat (% of energy)	30.2 ± 3.4	26.8 ± 3.2	27.2 ± 3.4	0.0001	26.9 ± 3.9	25.5 ± 4.3	24.6 ± 2.9	0.005
MUFA (% of energy)	11.3 ± 1.6	9.6 ± 1.4	9.7 ± 1.7	<0.0001	10.3 ± 1.7	9.5 ± 1.9	8.8 ± 1.4	<0.0001
PUFA (% of energy)	6.2 ± 0.7	6.1 ± 0.8	6.3 ± 0.8	0.55	5.8 ± 0.7	5.7 ± 1.0	5.8 ± 0.8	0.93
Carbohydrate (% of energy)	52.9 ± 4.2	56.1 ± 4.2	54.7 ± 4.4	0.08	52.9 ± 5.6	51.8 ± 6.4	54.1 ± 5.8	0.37
Alcohol (% of energy)	1.6 ± 2.8	1.3 ± 1.7	1.3 ± 2.3	0.54	4.9 ± 4.9	6.8 ± 6.4	5.3 ± 5.8	0.80
Vitamin E (mg/4184 kJ)	3.8 ± 0.7	4.0 ± 0.5	4.3 ± 0.7	0.0001	3.3 ± 0.5	3.5 ± 0.6	3.9 ± 0.6	<0.0001
Vitamin K (µg/4184 kJ)	89 ± 23	137 ± 36	164 ± 49	<0.0001	78 ± 16	106 ± 34	133 ± 31	<0.0001
Thiamine (mg/4184 kJ)	0.4 ± 0.1	0.5 ± 0.1	0.5 ± 0.1	<0.0001	0.4 ± 0.1	0.5 ± 0.1	0.5 ± 0.1	<0.0001
Riboflavin (mg/4184 kJ)	0.6 ± 0.1	0.7 ± 0.1	0.8 ± 0.1	<0.0001	0.6 ± 0.1	0.6 ± 0.1	0.7 ± 0.1	<0.0001
Niacin (mg/4184 kJ)	8.7 ± 1.5	9.0 ± 1.6	10.5 ± 2.0	<0.0001	8.8 ± 1.7	9.5 ± 1.5	9.9 ± 2.2	0.008
Vitamin B-6 (mg/4184 kJ)	0.6 ± 0.1	0.7 ± 0.1	0.8 ± 0.1	<0.0001	0.6 ± 0.1	0.7 ± 0.1	0.7 ± 0.1	<0.0001
Vitamin B-12 (µg/4184 kJ)	3.4 ± 1.3	4.3 ± 1.2	5.0 ± 1.8	<0.0001	3.2 ± 1.1	4.3 ± 1.5	5.0 ± 1.9	<0.0001
Folate (µg/4184 kJ)	154 ± 44	197 ± 34	233 ± 39	<0.0001	138 ± 31	168 ± 30	207 ± 51	<0.0001
Zinc (mg/4184 kJ)	4.2 ± 0.4	4.4 ± 0.4	4.6 ± 0.4	<0.0001	4.1 ± 0.5	4.2 ± 0.5	4.4 ± 0.5	0.003
Energy (kJ/d)	7598 ± 1458	7987 ± 1112	7499 ± 959	0.71	9793 ± 1845	10196 ± 1717	9666 ± 1692	0.75

NRF9.3, Nutrient-Rich Food Index 9.3; RAE, retinol activity equivalent; T, tertile. [a] In each season, a 4-day weighed dietary record was conducted: November and December 2002 (autumn), February 2003 (winter), May 2003 (spring) and August and September 2003 (summer). For each individual, the mean value of the 16-day data was used. Values are means ± standard deviations unless otherwise indicated. [b] Calculated based on general linear models. [c] A higher score indicates a higher diet quality.

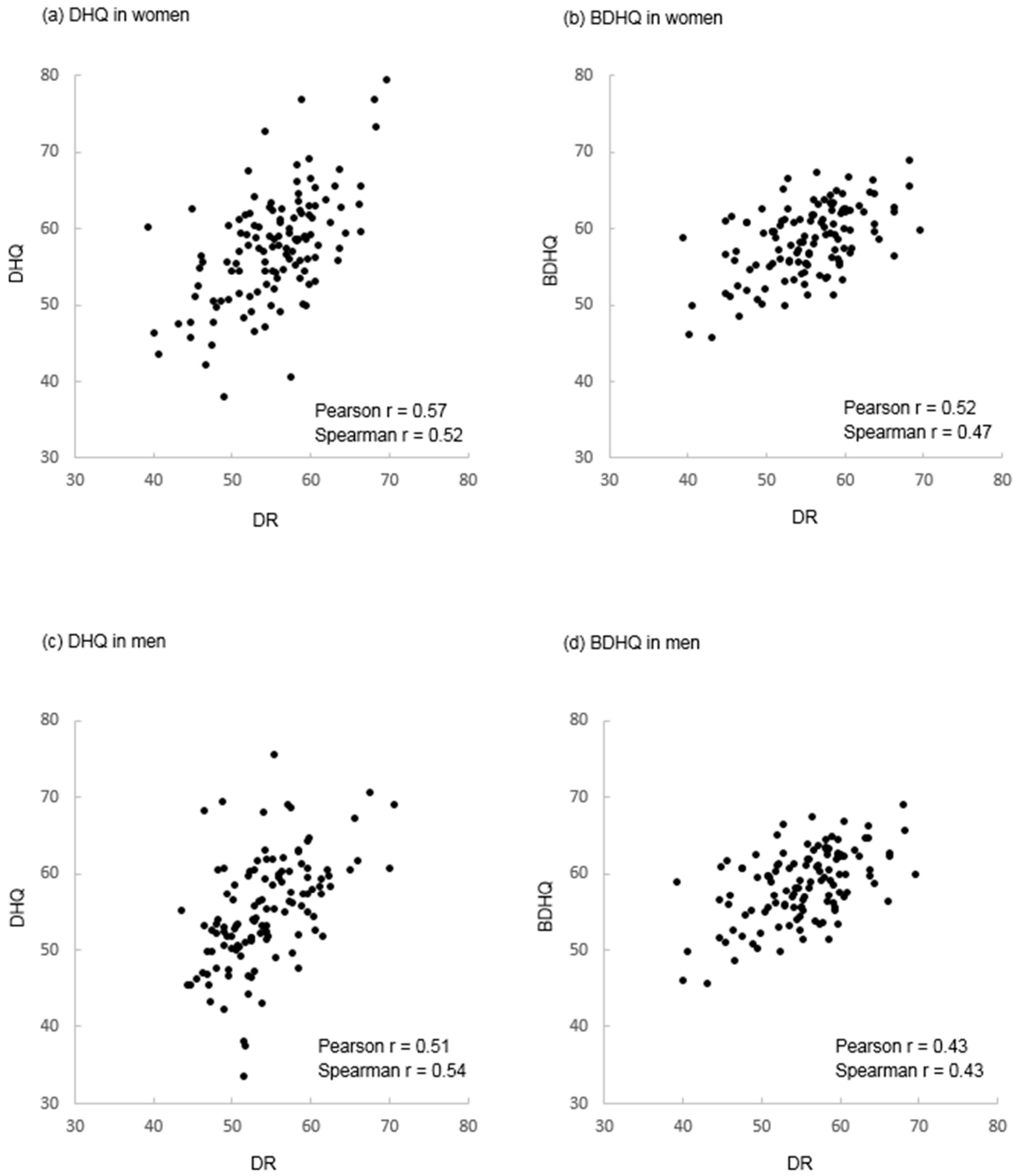

Figure S1. Relationship between the total score of the Healthy Eating Index-2015 estimated by a 16-day weighed dietary record (DR) conducted over a year and that estimated by a comprehensive diet history questionnaire (DHQ) or by a brief diet history questionnaire (BDHQ) in Japanese women (*n* 121) and men (*n* 121). In each season, a 4-day weighed DR was conducted: November and December 2002 (autumn), February 2003 (winter), May 2003 (spring) and August and September 2003 (summer). For each individual, the mean value of the 16-day data was used. The DHQ and BDHQ used in this analysis were conducted before the period of the DR (November 2002).

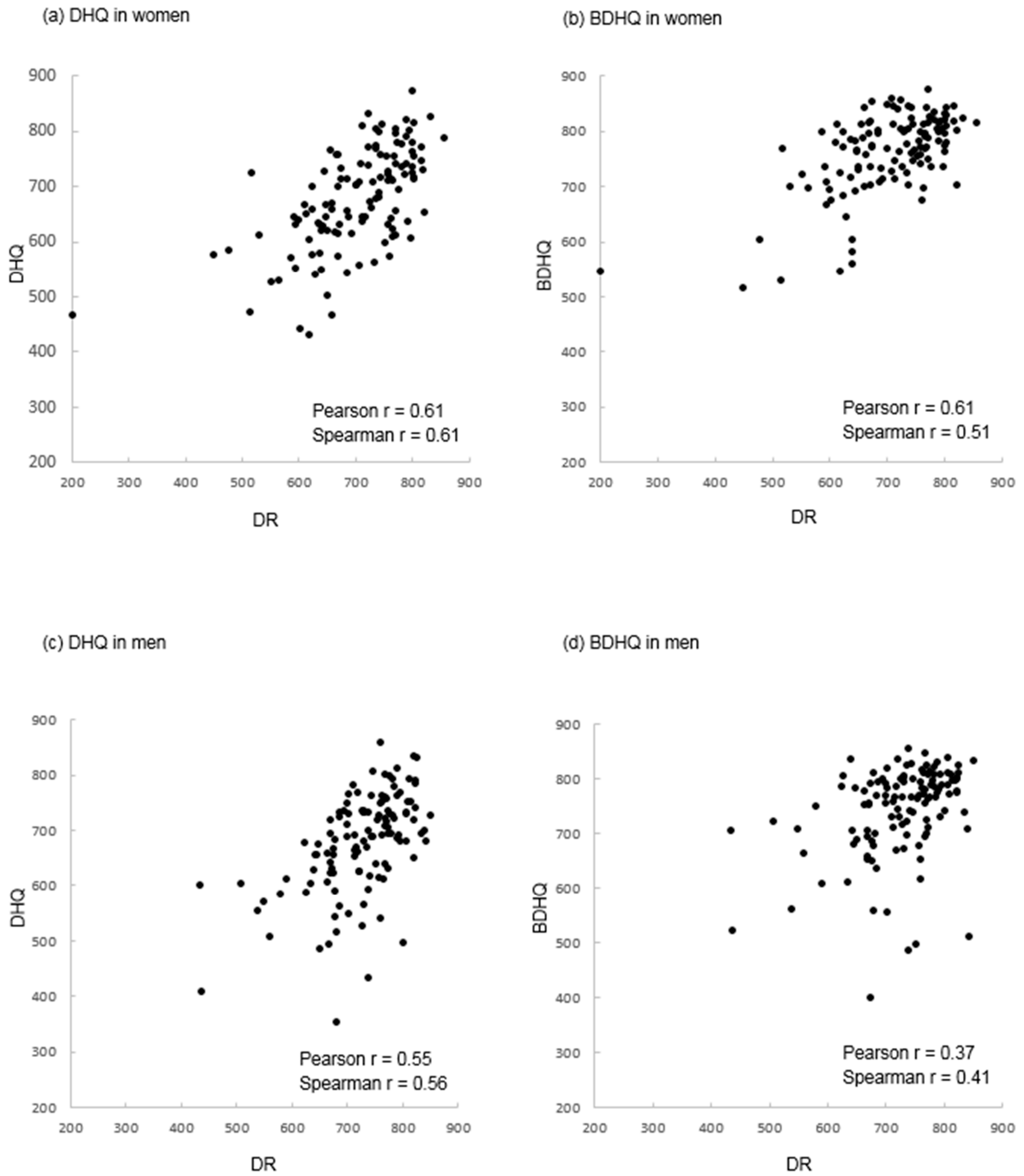

Figure S2. Relationship between the total score of the Nutrient-Rich Food Index 9.3 estimated by a 16-day weighed dietary record (DR) conducted over a year and that estimated by a comprehensive diet history questionnaire (DHQ) or by a brief diet history questionnaire (BDHQ) in Japanese women (*n* 121) and men (*n* 121). In each season, a 4-day weighed DR was conducted: November and December 2002 (autumn), February 2003 (winter), May 2003 (spring) and August and September 2003 (summer). For each individual, the mean value of the 16-day data was used. The DHQ and BDHQ used in this analysis were conducted before the period of the DR (November 2002).
